# Rapid evolution of *Medicago polymorpha* during invasion shifts interactions with the soybean looper

**DOI:** 10.1002/ece3.5572

**Published:** 2019-08-16

**Authors:** Chandra N. Jack, Maren L. Friesen

**Affiliations:** ^1^ Department of Plant Biology Michigan State University East Lansing Michigan; ^2^ BEACON Center for the Study of Evolution in Action East Lansing Michigan; ^3^ Department of Plant Pathology Washington State University Pullman Washington; ^4^ Department of Crop and Soil Sciences Washington State University Pullman Washington

**Keywords:** community ecology, invasive plants, *Medicago polymorpha*, plant–herbivore interactions, rapid evolution

## Abstract

The Enemy Release Hypothesis posits that invasion of novel habitats can be facilitated by the absence of coevolved herbivores. However, a new environment and interactions with unfamiliar herbivores may impose selection on invading plants for traits that reduce their attractiveness to herbivores or for enhanced defenses compared to native host plants, leading to a pattern similar to enemy release but driven by evolutionary change rather than ecological differences. The Shifting Defense Hypothesis posits that plants in novel habitats will shift from specialized defense mechanisms to defense mechanisms effective against generalist herbivores in the new range. We tested these ideas by comparing herbivore preference and performance of native (Eurasia)‐ and invasive (New World)‐range *Medicago polymorpha*, using a generalist herbivore, the soybean looper, that co‐occurs with *M. polymorpha* in its New World invaded range. We found that soybean loopers varied in preference and performance depending on host genotype and that overall the herbivore preferred to consume plant genotypes from naïve populations from Eurasia. This potentially suggests that range expansion of *M. polymorpha* into the New World has led to rapid evolution of a variety of traits that have helped multiple populations become established, including those that may allow invasive populations to resist herbivory. Thus, enemy release in a novel range can occur through rapid evolution by the plant during invasion, as predicted by the Shifting Defense Hypothesis, rather than via historical divergence.

## Graphical abstract

Our study found that the generalist herbivore, *Chrysodeixis includens* (soybean looper), preferred to feed on leaves from Eurasian native‐range *Medicago polymorpha* rather than from *M. polymorpha* that have recently invaded the New World, the range where the herbivore occurs. This preference for evolutionarily naïve genotypes shows that New World (invasive range) *M. polymorpha* populations underwent an evolutionary change in less than 500 years, which may have made it more similar to native, nonintroduced plants. This would result in a pattern similar to enemy release but occurring through contemporary evolution by the plant during invasion rather than historical divergence at the species or genus level.

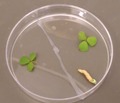

## 1 INTRODUCTION

Understanding the mechanisms that allow exotic species to become invasive is key to limiting invasive species that are already present and prevent future exotics from becoming established. Ecological interactions as a driving force for the success or failure of the establishment of exotics is a concept derived from early biocontrol studies (DeLoach, 1991; McFadyen, 1998; Smith & van den Bosch, 1967). The successful establishment of an invading plant may depend on its phylogenetic similarity to native plants, the similarity of native herbivores to those from its home range, and how quickly it adapts to its novel environment (Harvey, Nipperess, Britton, & Hughes, 2013). Rapid evolution in plant populations may lead to traits that are more similar to plants in their current environment than to plants from their native range (Hawkes, 2007). Biogeographic comparisons of plants can point to genetically determined changes in a plant's traits after it has evolved in an unfamiliar environment, compared to its native habitat. These traits can influence the outcome of interactions with local enemies and may be key to explaining why we see such biogeographic variation in plant performance (Parker & Gilbert, 2007).

Classical theories relating species invasion to host–enemy interactions assume historical coevolutionary processes that drive strictly ecological processes. According to Ehrlich and Raven (1964), coevolutionary studies provide a starting point for understanding community evolution and ecology, especially as these interactions are likely to have played a key role in species diversity in plants and insects. Several competing ecological theories have been developed to predict the successful establishment of introduced plants based on the response of native herbivores to these exotics. The most prevalent theory is known as the Enemy Release Hypothesis (ERH), which predicts that native herbivores will prefer to feed on plants from their native range and that they will have better growth rates on these plants. Under this theory, exotic plants have greater success in their new environment because they have left behind coevolved natural enemies and because native generalist herbivores have a greater impact on native plants than on these exotics (Keane & Crawley, 2002; Schaffner et al., 2011). Conversely, the Biotic Resistance Hypothesis (BRH) posits that native herbivores would limit the range expansion of exotic plants due to their preference and higher growth rates on evolutionarily naïve plants (Parker & Hay, 2005). In contrast, the Novel Weapons Hypothesis (NWH) posits that invasive plant defense systems will be more effective in novel interactions with herbivores (Callaway et al., 2008; Callaway & Ridenour, 2004; Schaffner et al., 2011; Zheng et al., 2015).

However, predictions about invasive plant success based solely on these ecological theories fail to consider rapid evolution as a mechanism leading to their establishment. Several studies have found that herbivory is a strong selective force on plant defensive traits. Plants can rapidly adapt to their environment as herbivores drive ecological and evolutionary changes in plant populations quickly leading to increased survival in their new habitat (Agrawal, Hastings, Johnson, Maron, & Salminen, 2012; Zangerl & Berenbaum, 2005; Zangerl, Berenbaum, & Mallet, 2003; Züst et al., 2012). If exotic plants do leave behind enemies, this may lead to changes in plant antiherbivore defenses. Resistance traits in particular are costly to make. A lack of herbivorous insects could lead to the evolution of reduced phytochemical production and the channeling of those resources toward growth, known as the Evolution of Increased Competitive Ability (EICA; Agrawal et al., 2012; Ali & Agrawal, 2012; Blossey & Notzold, 1995; Uesugi & Kessler, 2013) Müller‐Schärer, Schaffner, and Steinger (2004) argue that moving to a new location releases plants from some natural enemies, mostly specialists; but changing environments does not lead to a decrease in selective pressure on defense traits but rather changes them from focusing on both specialists and generalists to mainly focusing on generalist herbivores. Under this theory, known as the Shifting Defense Hypothesis (SDH), there should be selection for an increase in defensive toxins, which are more likely to affect nonadapted generalists (Doorduin & Vrieling, 2011). Lankau found that removing the dominant specialist of the invasive black mustard, *Brassica nigra* increased the concentration of the defensive compound sinigrin, which significantly deterred generalist herbivores, but removing the dominant generalist led to intermediate sinigrin concentrations (2007). Evidence for the SDH has also been shown in the California poppy (Leger & Forister, 2005), in *Arabidopsis* (Züst et al., 2012), and in *Senecio pterophorus* (Castells, Mulder, & Pérez‐Trujillo, 2014). These studies not only provide evidence of contemporary selection and the rapid evolution of plant defensive traits in response to herbivore interactions but also show that evolutionary processes can have an effect on ecological process.

Plants have developed secondary metabolites that have a crucial role in both direct and indirect defenses against herbivores (Orians & Ward, 2010; War et al., 2012). While these defenses include both constitutive and inducible defenses, inducible defenses that are only activated after insect attack are especially interesting because it requires the plant to recognize the attacking insect and produce effective defensive compounds (Fürstenberg‐Hägg, Zagrobelny, & Bak, 2013; Woodard, Ervin, & Marsico, 2012). Both the Enemy Release and Biotic Resistance Hypotheses rely on evolutionary unfamiliarity between interacting species, a concept known as evolutionary mismatching, which can occur through two different mechanisms (Verhoeven, Biere, Harvey, & Putten, 2009). The first described as an elicitor‐receptor mechanism (Kniskern & Rausher, 2001) prevents the plant from recognizing molecules produced by the insect, which hampers the plant's ability to mount a defense and may suppress invasion. The second mechanism, through toxin‐detoxifier systems, is where plants evolve toxic secondary metabolites. If plants are introduced to a new environment, they may become established not because they have left behind their enemies but because native generalist herbivores avoid and have a reduced performance on these exotic plants because of their unfamiliar defensive compounds (Novel Weapons Hypothesis; Callaway et al., 2008; Callaway & Ridenour, 2004; Schaffner et al., 2011; Zheng et al., 2015). The EICA and SDH hypotheses both rely upon contemporary evolution and could occur through the toxin‐detoxifier system.

The medics are a group of legume species that are indigenous to the Mediterranean Basin (Bena, Prosperi, Lejeune, & Olivieri, 1998). *Medicago polymorpha* is one of the most common medic species outside cultivated alfalfa (*Medicago sativa*) and is now considered invasive (Paredes et al., 2002; Small & Jomphe, 1989). It was purposefully introduced to North and South America and Australia in the 1800s and has spread worldwide (de Haan & Barnes, 1998; Lesins & Lesins, 1979; Small & Jomphe, 1989; Spira & Wagner, 1983). *Medicago polymorpha* may have been accidentally introduced much earlier with records of its presence in S. America dating back 450 years (Del Pozo, Ovalle, & Avendaño, 1989). Previous studies of plant–herbivore interactions using medics have mainly focused on the direct and indirect effects the presence of an invasive species has on native host plants or, have been field studies so only ambient herbivory was measured (Lau, 2012; Lau & Strauss, 2005; Leakey & Lau, 2012). Those studies have in some instances shown evidence of genotypic differences between the ranges but are not direct assessments of how herbivores respond to Eurasian genotypes versus those from the New World.

**Figure 1:**
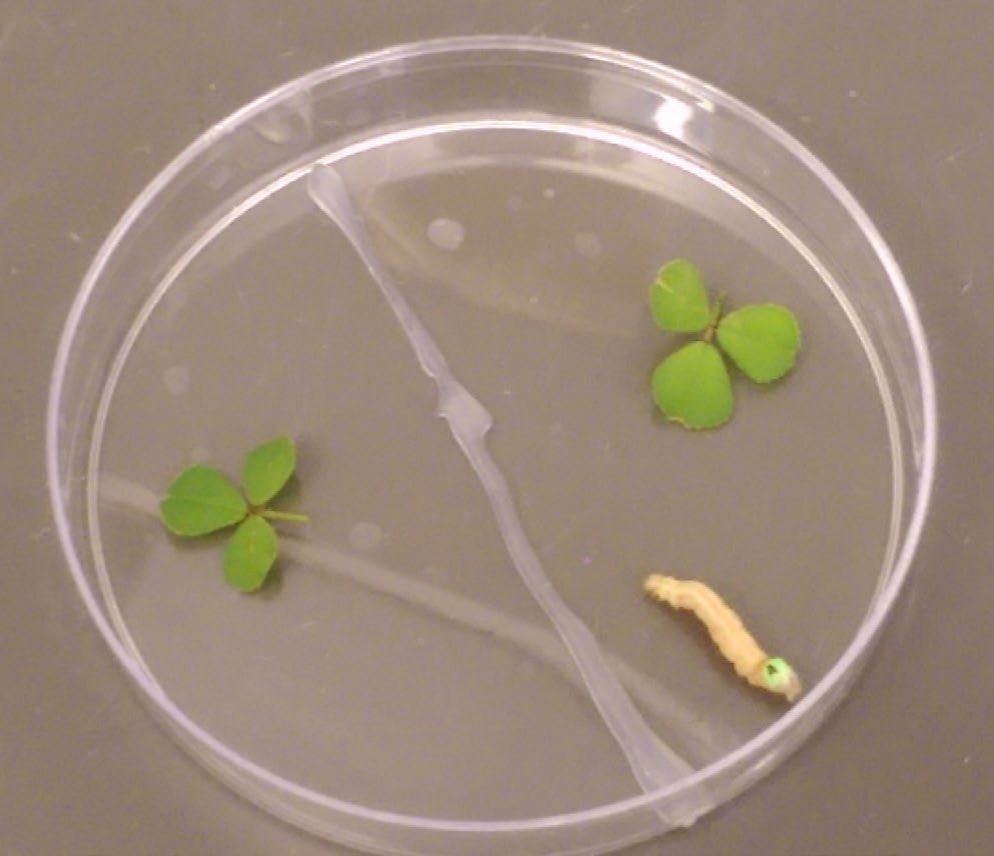
Picture of *Medicago polymorpha* and the soybean looper setup for the preference assays

**Figure 2:**
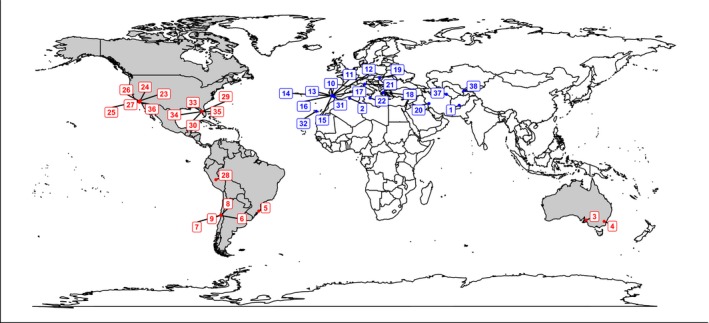
Distribution of *Medicago polymorpha* genotypes and soybean looper range. The map shows the locations of the New World invasive‐range (red) and Eurasian native‐range (blue) *M. polymorpha* genotypes used in the experiment. The countries shaded in gray represent the range of the soybean looper, which overlaps with the invasive‐range *M. polymorpha*

In this study, we measure the feeding preference and growth of a common generalist herbivore, the soybean looper (*Chrysodeixis includens*), when allowed to feed on *M. polymorpha* genotypes originating from contrasting ranges: *M. polymorpha*'s native range (Eurasia) and its invasive range (the New World, which overlaps with the herbivore; Figures 1 and 2). If *Medicago* populations underwent rapid evolutionary changes due to novel biotic interactions, then some of those changes may have allowed it to successfully establish in their new environment and avoid herbivory from insects present in that range. Specifically, we predict that *Medicago* populations in the New World will have evolved during the course of invading their novel habitat in ways that result in them interacting less strongly with generalist herbivores than naïve populations from their native range around the Mediterranean. This result would demonstrate evidence of rapid evolution in the introduced populations of *M. polymorpha*, which could be due to interactions with diverse herbivores in the invaded range, selection imposed by other novel biotic or abiotic stresses, or nonadaptive evolutionary processes such as genetic drift during founder events.

## 2 MATERIALS AND METHODS

### 2.1 *Medicago polymorpha* germplasm, germination, and growth

*Medicago polymorpha* is an annual legume that forms a symbiotic relationship with the rhizobium *Ensifer medicae* and is highly selfing in nature. We used 19 genotypes of *M. polymorpha* from their invasive range (North America, South America, and Australia,) and 19 genotypes from their native range (Eurasia) obtained from both the USDA NGRP and personal field collection (Table 1). The field collection was created by collecting individual pods from the ground at least 1 m apart. These pods were assumed to represent unique maternal lineages because *M. polymorpha* is highly selfing in nature and the pods rarely disperse large distances (Lesins & Lesins, 1979). All parental plants of the genotypes we used were started from a single pod, selfed from a single progeny for three generations and grown in a common‐garden greenhouse environment to control for maternal effects. Seeds from these plants were used in our experiment. This method of developing a seed collection does not lead to artificial inbreeding in highly selfing plants such as *M. polymorpha*. Previous work on field levels of heterozygosity in this species found no evidence of inter‐accession crossing, and only 1 of the 4 accessions tested showed low levels (0%–4%) of intra‐accession crossing when they attempted to simulate a natural environment in a greenhouse (Vitale, Pupilli, Labombarda, & Arcioni, 1998). We scarified *M. polymorpha* seeds to break their physical dormancy by rubbing the seeds with 600‐grade sandpaper. The seeds were then sterilized in 6% commercial bleach for 3 min to remove any external bacteria. After sterilization, the seeds were imbibed in dH_2_O for three days at 4°C in the dark to stratify. We removed the seeds from the cold and placed them in a dark cabinet overnight. The germinated seedlings were grown from May 28, 2015, to June, 15, 2015, in a plant growth room (conditions: 16‐hr days at 22°C) in a sterile soil substrate (2 part Suremix™: ½ part sand) before being transplanted to 656 ml D‐pots (Stuewe & Sons) and grown in a greenhouse from June, 15, 2015, to July 13, 2015 (Lat: 42.723°; Long: −84.473°; conditions: 16‐hr days with temperature range of 18°C−24°C).

**Table 1:** Genotypes used in herbivore assays

Map ID	Genotype	Country	Population	Latitude	Longitude	Range
1	PI250782	Afghanistan	MP‐PI250782	31.6166667	65.7166667	Native
2	W65435	Algeria	MP‐W65435	35.7	2.6	Native
3	PI197336	Australia	PI197336	−34.533333	138.733333	Invasive
4	W65527	Australia	MP‐W65527	−35.3	149.133333	Invasive
5	PI404356	Brazil	MP‐PI404356	−29.35	−49.733333	Invasive
6	PI368939	Chile	MP‐PI368939	−31.9	−71.5	Invasive
7	PI368940	Chile	MP‐PI368940	−31.7	−71.65	Invasive
8	PI368950	Chile	MP‐PI368950	−31.61	−71.53	Invasive
9	PI368959	Chile	MP‐PI368959	−31.625556	−71.524167	Invasive
10	CMS 12‐4	Portugal	Portugal1	37.226417	−7.438861	Native
11	CMNS 4‐8	Portugal	Portugal1	37.226417	−7.438861	Native
12	CMNS 5‐2	Portugal	Portugal1	37.226417	−7.438861	Native
13	GILNS 1‐2	Portugal	Portugal2	37.110472	−7.650417	Native
14	GILNS 4‐1	Portugal	Portugal2	37.110472	−7.650417	Native
15	GILNS 10‐6	Portugal	Portugal2	37.110472	−7.650417	Native
16	GILNS 11‐4	Portugal	Portugal2	37.110472	−7.650417	Native
17	GILNS 13‐6	Portugal	Portugal2	37.110472	−7.650417	Native
18	W65319	Greece	MP‐W65319	39.0666667	21.9833333	Native
19	W65565	Hungary	MP‐W65565	47.3333333	19.8833333	Native
20	PI227025	Iran	MP‐PI227025	32.63	48.26	Native
21	W65256	Italy	MP‐W65256	43.8	11.2833333	Native
22	W65375	Malta	MP‐W65375	35.8666667	14.3666667	Native
23	Mt. Wilson‐2	USA (CA)	California1	34.2238	−118.0616	Invasive
24	Mt. Wilson‐4	USA (CA)	California1	34.2238	−118.0616	Invasive
25	NM58‐12	USA (CA)	California2	33.970999	−118.43676	Invasive
26	NM58‐13	USA (CA)	California2	33.970999	−118.43676	Invasive
27	NM58‐35	USA (CA)	California2	33.970999	−118.43676	Invasive
28	PI308523	Peru	MP‐PI308523	−11.25	−74.41	Invasive
29	Rivercrest‐1	USA (FL)	Florida2	27.9894833	−82.4658	Invasive
30	Rivercrest‐11	USA (FL)	Florida2	27.9894833	−82.4658	Invasive
31	W65325	Spain	MP‐W65325	36.5333333	−6.3	Native
32	W65390	Spain	MP‐W65390	28.15	−16.633333	Native
33	St. Augustine‐11	USA (FL)	Florida1	27.9894833	−82.4658	Invasive
34	St. Augustine‐14	USA (FL)	Florida1	27.9894833	−82.4658	Invasive
35	St. Augustine‐3	USA (FL)	Florida1	27.9894833	−82.4658	Invasive
36	Starlight	USA (CA)	California3	33.3879	−118.416	Invasive
37	W62449	Turkmenistan	MP‐W624449	37.9667	58.3333	Native
38	W68297	Uzbekistan	MP‐W68297	40.0666667	68.4166667	Native

### 2.2 Soybean looper larvae (*C. includens*)

The soybean looper is a generalist herbivore whose hosts include field crops such as soybeans, tomatoes, and peanuts, and wild plants such as the common cocklebur and sow thistle. Third instar soybean loopers, obtained from Benzon Research, were used in all assays. The loopers were fed a multispecies artificial diet from Southland Products Inc. until they were used in the experiment.

### 2.3 Food preference assay

We used 38 *M. polymorpha* genotypes in 84 pairings between plant ranges (one native‐range genotype with one invasive‐range genotype; see Appendix 1). The original plan was to compare preference between all possible combinations of native‐ and invasive‐range genotypes, but we became tissue‐limited during experimental setup. We then randomly chose pairings, which is why some genotypes occur more than others, but all pairs are only represented five times in the dataset to prevent overinfluence of a specific pairing. By setting up the experiment in this manner, we are able to examine range effects on preference but not genotypic variation. We placed an equal amount of native‐range or invasive‐range leaf material (~30 mg) on each side of a 100‐mm petri dish. One caterpillar was placed in the middle of the petri dish and left to feed for 24 hr. Each pairing had 5 replicate petri dishes. The dishes were placed in large plastic bins with moist paper towels to prevent the leaves from drying out. Petri dishes containing only leaves (~25 mg on average) of the genotypes served as controls to account for changes in leaf weight due to water loss. After 24 hr, we reweighed the remaining leaf material. The amount of leaf tissue consumed of each genotype was divided by the total amount of food consumed within the petri dish. Values over 0.5 indicate more of that genotype was preferred over the other available genotype and were given a score of 1, and the nonpreferred genotype was given a score of 0.

### 2.4 Insect performance assays

We measured the growth rate of larvae on intact *M. polymorpha* plants to test the nutritional suitability of the plants. Six‐week‐old plants were transferred from their D‐pots into individual 12.7 cm × 12.7 cm plastic boxes and misted with dH_2_O. We weighed five soybean loopers of approximately the same size and placed them in each plastic box with the plant. Each genotype was replicated five times. After one week, we collected the caterpillars and reweighed them. 35% of the caterpillars were either dead or unaccounted for and presumed dead. They were excluded, and an average weight was calculated of the living caterpillars. Prior to our experimental setup, we tested several containment systems to prevent escape as required by our USDA plant pest permit (P526P‐ 15‐00942). Given enough time, the larvae are able to chew through Styrofoam, so we chose hard plastic deli boxes that come with lids that crimp on both sides of the box walls. The lids were sealed on three sides, but not the fourth to allow air exchange while preventing escape. In a previous experiment, we weighed 125 caterpillars, dried them in an oven for three days at 55°C, and then reweighed them to find an equation for estimating dry weight from fresh (Dry weight = 0.114 × Fresh weight + 0.1992; *R* ^2^ = .514). We calculated the estimated initial and final dry weights of the caterpillars for this experiment using this equation and used these values to calculate the relative growth rate (RGR) based on our calculation of the mean daily weight [((# Days of experiment × Initial Larvae Weight) + Factorial(# Days of experiment) * Average weight change per day))/ # Days of experiment] and following the procedure described by Waldbauer (1968). We also measured the mortality rate of the soybean loopers on each plant. It is unlikely that RGR or mortality rate was influenced by cannibalism because the experiments were not conducted under conditions that encouraged it (e.g., overcrowded containers, limited food source, and late stage larvae).

### 2.5 No‐choice assay

We compared the amount of leaf material consumed by the soybean loopers when they were not given a choice for 10 of the genotypes used in the full experiment. This experiment used the same set of plants but was set up one week after the choice experiment. Soybean loopers, approximately the same size as the ones that were used in the preference experiment, were individually placed in six‐well tissue culture plates (Corning‐ Cat #3516) and given ~30 mg of leaf material from only one genotype. Leaf tissue was reweighed after 72 hr.

### 2.6 Statistical analyses

Data analysis was done using R version 3.5.2 (R Core Team, 2018). Data figures were created using ggplot2 2.0.0 (Wickham, 2009). The map was created using packages: ggplot2, OpenStreetMap (Fellows, 2016), maps (Becker, Wilks, Brownrigg, Minka, & Deckmyn, 2018), and ggrepel (Slowikowski, 2018).

Range was treated as a fixed effect in all models. We ran exploratory analyses to determine whether the absolute value of latitude had any explanatory power, but model selection using the likelihood ratio test found the models to be a better fit when it was not included. Genotype was included as a random variable nested within range to account for differences between genotypes not represented by the range term, and population was included as a random variable to account for the spatial aggregation of some genotypes within our sampling design. The population term thus accounts for any nonindependence due to genetic similarity of host genotypes originating from the same population. Pair was included to control for nonindependence of the genotypes within petri dishes for the preference assay. Normality, overdispersion, and heteroscedasticity of residuals were checked on all models where appropriate using DHARMa (Hartig, 2019). The preference data were analyzed using a generalized linear mixed model with a binomial distribution with the lme4 package (Bates, Mächler, Bolker, & Walker, 2014). To analyze the data from the insect growth assay, we removed three data points that represented larvae that lost body weight greater than two standard deviations of the mean over the course of the experiment, as these caterpillars were almost certainly not healthy prior to the start of the experiment. Then, we analyzed RGR using a linear mixed model with the lme4 package. Caterpillar mortality based on a count of the number of dead or missing caterpillars was analyzed using a generalized linear mixed model with a Poisson distribution. Not collapsing missing caterpillars into the dead column may have given a better model to analyze mortality but we did not record when we found dead caterpillars versus when they were just missing in this experiment. This decision was made based on a small pilot experiment where we did record alive caterpillars versus intact dead bodies versus missing caterpillars. Before declaring a caterpillar missing, all areas around the top layer of the soil were inspected, and then, plants were removed from their containers for a closer inspection of the soil. Several times, we found remnants of what were either decomposed bodies or molted skin, which could not be categorized with certainty. To analyze the no‐choice consumption data, the proportion of leaf tissue eaten by the 10 genotypes when alone versus when paired was analyzed using a generalized linear mixed model with a beta distribution using glmmTMB (Brooks et al., 2017). An initial check of the residuals revealed heteroscedasticity, which was then built into a new model. Comparison of the two models did not show a significant difference (*χ* ^2^ = 0.911, *p* = .3398), but the second model was used for analysis. Fixed effects were analyzed using likelihood ratio tests for all models.

## 3 RESULTS

### 3.1 Native‐ and invasive‐range genotype herbivore choice experiments

**Figure 3:**
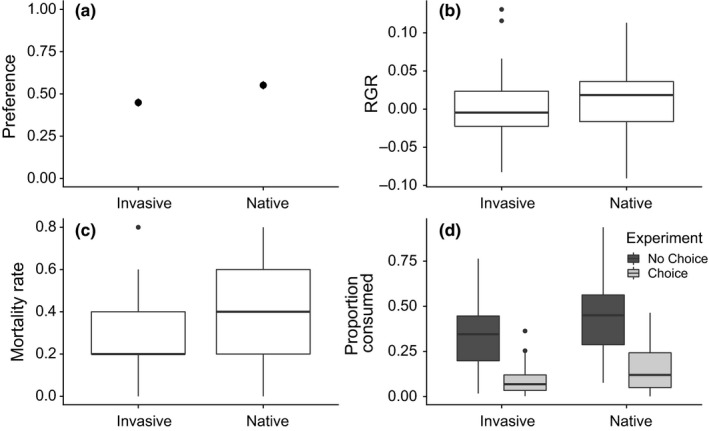
Soybean looper preference and performance assays on *Medicago polymorpha*. (a) The herbivore preference experiment showed that the native‐range Eurasian *M. polymorpha* genotype was preferred in 55.1% of the trials. (b) Relative growth rate (RGR) of the soybean loopers and their (c) mortality were higher but not significantly different on genotypes from Eurasia. (d) Tissue consumption of the 10 genotypes used in the no‐choice assays was higher for native‐range genotypes with and without the presence of a second genotype. The dot plots show mean plus standard error while the boxplot whiskers extend to lowest and highest values that are within 1.5 × IQR of the hinges

Feeding preference of the soybean looper was significantly influenced by the range from which the *M. polymorpha* host genotypes originated (*p* = 0.047; Table 2 and Figure 3a). The native‐range host genotypes (Eurasian) were preferred in 55.1% (±2.4%) of the trials over the co‐occurring New World genotypes.

**Table 2:** Analysis of herbivore preference and performance

Effect of range on	*χ* ^2^	*p*‐Value
Preference	3.9596	.047*
Insect RGR	1.8558	.173
Insect mortality	3.2523	.071●

* *p* < .05;

^●^ .05 < *p *< .10.

### 3.2 Caterpillar growth and mortality on intact plants

The growth rate of the caterpillars is a performance metric that indicates the suitability of the plant. Although the soybean loopers tended to have a higher growth rate on Eurasian *M. polymorpha*, it was not significantly different from their growth rate on invasive New World tissue (Eurasian MP: 1.24 × 10^−2^ mg/day ± 5.29 × 10^−3^ mg/day; New World MP: 9.77 × 10^−4^ mg/day ± 4.32 × 10^−3^ mg/day, *p* = .183; Table 2 and Figure 3b). The number of caterpillar deaths was slightly higher on Eurasian range plants, but statistical significance was marginal (Eurasian MP: 1.88 deaths/plant ± 0.14; New World MP: 1.48 deaths/ plant ± 0.12, *p* = .071; Table 2 and Figure 3c).

### 3.3 No‐choice leaf consumption

We compared the proportion of leaf material eaten by the soybean loopers when they were only given either Eurasian or New World *M. polymorpha* leaves and analyzed whether consumption changed in the presence or absence of another food choice. The proportion of leaves from plants consumed from the Eurasian was higher than the proportion of invasive‐range leaf material regardless of the presence of another choice (Table 3 and Figure 3d).

**Table 3:** Analysis of herbivore tissue consumption when alone versus when paired for subset of genotypes

Tissue consumption affected by	*χ* ^2^	*p*‐Value
Range	6.3137	.006*
Choice–No Choice	249.2810	<.001*
Range: CNC	0.4981	.374

* *p* < .05.

## 4 DISCUSSION

The ability of exotic plants to become invasive upon establishment in a novel environment is puzzling, and current studies do not provide a clear consensus over the role of herbivorous insects in their establishment (Bezemer, Harvey, & Cronin, 2014; Chun, Kleunen, & Dawson, 2010; Harvey et al., 2010). While evolution is often thought a slow process that takes thousands of years, novel interactions between species can lead to rapid evolution that affect ecological dynamics (Johnson, Vellend, & Stinchcombe, 2009; Ohgushi, 2016; Slobodkin, 1980; Thompson, 1998, 1999; Turcotte, Reznick, & Hare, 2011). This is especially true in the case of antagonistic plant–insect interactions where there may be a continual defense‐counter response arms race. In our study, we compared the preference of a generalist herbivore, the soybean looper (*C. includens*), for native‐range (Eurasian) *M. polymorpha* populations versus New World host populations that have evolved in the same range as the looper, but for less than 500 years (invasive‐range *M. polymorpha*). We found that the soybean loopers showed a significant preference for the Eurasian *M. polymorpha* over the New World *M. polymorpha* when they were given a choice of leaf tissue (Figure 3a). We also compared the amount of leaf tissue that the soybean loopers ate when they were not given a choice versus when they were given a choice for a subset of the plants. In both cases, the soybean loopers showed a higher rate of consumption for Eurasian *M. polymorpha* genotypes, that is, the genotypes that did not have range overlap with the herbivore.

One of the problems with attempting to apply ecological theories such as the Enemy Release Hypothesis (ERH) or Biotic Resistance Hypothesis (BRH) to answer questions about established invasive plants is that those theories ignore evolutionary changes, unless the plant is newly established. Carthey and Banks (2012) ask when does an alien become a native species and how much exposure to novel enemies is enough for an exotic to lose its novelty. Plants can rapidly adapt to their environment as herbivores drive ecological and evolutionary changes in plant populations in their new habitat (Agrawal et al., 2012; Züst et al., 2012). Both Hawkes (2007) and Schultheis, Berardi, and Lau (2015) found that enemy release declined over time as native and introduced species coevolved, to where, approximately 150 years after introduction, invasive species have similar responses to herbivory as native community members. It is possible that something comparable happens with biotic resistance. It highlights the importance of knowing the range of the focal plants used in experiments as well as the distribution of the herbivores used in the assays. *M. polymorpha* has been a very successful invader, especially compared to other medic species (Bena, Lyet, Huguet, & Olivieri, 2005), which is suggestive of enemy release as a means for their expansion. However, introduced *M. polymorpha* genotypes have been present in their invaded range (Del Pozo et al., 1989; de Haan & Barnes, 1998; Spira & Wagner, 1983) between 200 and 500 years, allowing sufficient time to adapt to native herbivores such as the soybean looper, and other environmental factors (Agrawal et al., 2012; Lankau, 2007; Züst et al., 2012). When exotic *M. polymorpha* first arrived in the introduced range, it may have been susceptible to herbivore attack but over subsequent generations, it evolved to be less susceptible either through genetic drift or natural selection. Alternatively, it is possible that only better defended *M. polymorpha* genotypes were able to establish in the New World invaded range, resulting in evolution through lineage sorting. Any of these processes would yield a pattern similar to enemy release but achieved through evolutionary processes. Our results suggest that novel genotypes, particularly those from regions above southern Portugal (Helliwell et al., 2018), attempting to colonize would have their establishment hampered compared to already present *M. polymorpha*. We did not compare herbivory in wild populations under natural field conditions and thus are not making inferences about the impact of herbivory on traits in each range. Instead, we investigated whether there are heritable differences between the native‐ and invasive‐range genotypes of *M. polymorpha* in how these plants express traits that impact the ways in which they interact with herbivores. Our work highlights an evolutionary shift by *M. polymorpha* during invasion of the New World that has influenced the outcome of interactions with herbivores. It indicates that at least one plant trait potentially has evolved in the invaded range in a manner that influences the herbivore's preferences.

The difference in host preference and performance by the soybean looper could be an indication of the evolution of herbivore defense in *M. polymorpha* where changes in defense strategies are based on risk of herbivore attack (Orians & Ward, 2010). If herbivores are present but rarely attack in the invaded range, possibly because of differences in food cues (Stutz, Croak, Proschogo, Banks, & McArthur, 2017) or the presence of a more nutritional food source, then a shift from constitutive defenses toward inducible defenses might be expected. While nonsignificant, our results showed a trend toward higher growth rates on the Eurasian (native range) *M. polymorpha* genotypes, which in conjunction with the preference and no‐choice consumption data suggest a shift toward inducible defenses. If escape is only from generalist herbivores (Müller‐Schärer et al., 2004), then overall defensive compounds against generalists would be either maintained or increased (Doorduin & Vrieling, 2011; Orians & Ward, 2010) in New World (invaded range) plant genotypes. The slightly increased mortality on Eurasian native‐range *M. polymorpha* is puzzling but herbivore preference does not always fall in line with performance metrics (Ikonen, Tahvanainen, & Roininen, 2002; Orians et al., 1997; Tomas, Box, & Terrados, 2011), which may be due to differences in selection on traits related to performance versus preference (Orians et al., 1997). Another explanation is that there has been a change in the type or quantity of defensive compound produced. In this study, we only measured RGR, but did not analyze nutritional indices, which may have further illuminated shifts in secondary metabolites or differences in nutritional quality. If we had included efficiency of conversion of ingested food into biomass (ECI) and efficiency of conversion of digested food into biomass (ECD), it would allow us to determine whether there was a shift from compounds that promote toxicity to those that are more antinutritional or antidigestive (Chen, 2008). In the related species, *M. sativa* (alfalfa) constitutive defense is mainly based on saponins and tannins but different varieties show variation in defensive proteins (Agrell, Oleszek, Stochmal, Olsen, & Anderson, 2003; Ramirez & Spears, 2014). Biochemical assays are now in place that will allow quantification of various defensive compounds in *M. polymorpha* to answer those questions (Jack, Rowe, Porter, & Friesen, 2019).

A recent study by Helliwell et al. (2018) found that the origins of the invaded range populations can be traced back to populations from a small region in the native Eurasian range that spans from northern Morocco to Southern Portugal. Population genetic analysis of the native range found *M. polymorpha* genotypes could be divided into two clusters, only one of which was represented in the invaded range indicating a genetic bottleneck that reduced genetic diversity (Total gene diversity—native range (Ht = 0.094) versus the invaded range (Ht = 0.057; Helliwell et al., 2018). In another plant system, even though the expected heterozygosity of introduced populations of Canary Island St. John's wort was half that of native populations, it still showed adaptation to the local environment (Dlugosch & Parker, 2008). Host shifts of native lepidopteron to a related introduced species, *M. sativa* (alfalfa), have been reported and found to be associated with genetic changes in 200 years (Graves & Shapiro, 2003; Nice, Fordyce, Shapiro, & Ffrench‐Constant, 2002). Despite low overall survival of *M. polymorpha* in the introduced California range (from 0.025% to 6.3%), there is some evidence of variation in the average fitness of genotypes in response to herbivory (terHorst & Lau, 2015), indicating the potential for rapid evolution in this species. We sampled as many populations as possible and included replicate genotypes for as many populations as were available. This hierarchical sampling accounts for range, between‐population, and within‐population genetic variation and is captured in the mixed models we used for analysis. The study by Helliwell et al. (2018) based on a novel, expanded collection that was not available to us showed that *M. polymorpha* variation is highest at the population level and lowest at the range level and found evidence supporting rapid evolution for flowering time in the invaded region. Future work leveraging genetic and genomic tools could partition how much of the change in plant–herbivore interactions is due to adaptation through natural selection versus genetic drift (Agrawal et al., 2015; Keller & Taylor, 2008; Schrieber et al., 2017). Thus, although there are alternative evolutionary hypotheses for the shift in plant–herbivore interactions that we document, this does not change our main assertion that there has been an evolutionary change, be it via adaptation through natural selection or evolution through genetic drift, resulting in New World genotypes rapidly evolving to have traits that impact their interactions with herbivores.

## 5 CONCLUSION

Our study found that the generalist herbivore, *C. includens* (soybean looper), preferred to feed on leaves from Eurasian native‐range *M. polymorpha* rather than from *M. polymorpha* that have recently invaded the New World, the range where the herbivore occurs. This preference for evolutionarily naïve genotypes shows that New World (invasive range) *M. polymorpha* populations underwent an evolutionary change in less than 500 years, which may have made it more similar to native, nonintroduced plants. This would result in a pattern similar to enemy release but occurring through contemporary evolution by the plant during invasion rather than historical divergence at the species or genus level.

## CONFLICT OF INTERESTS

All authors declare they have no conflict of interest.

## AUTHOR CONTRIBUTIONS

CNJ and MLF conceived and designed the experiment and wrote the manuscript. CNJ performed the experiment and analyzed the data.

## Data Availability

All data and R scripts used to analyze data are available at Dryad Digital Repository (https://doi.org/10.5061/dryad.84h07s1).

## APPENDIX 1

**Table A1:** Number of pairs that included each genotype. Size of the experiment precluded using all native–invasive pair combos

AfghanistanPI250782	AlgeriaW65435	AustraliaPI197336	AustraliaW65527	BrazilPI404356
3^a^	7^a^	7	9^a^	4^a^
ChilePI368939	ChilePI368940	ChilePI368950	ChilePI368959	CMNS4_8
1^a^	6	3	3	1
CMNS5_2	CMS12_4	GILNS1_2	GILNS10_6	GILNS11_4
2	8	3	9	6
GILNS13_6	GILNS4_1	GreeceW65319	HungaryW65565	IranPI227025
3	6	4^a^	5	3
ItalyW65256	MaltaW65375	Mt.Wilson_2	Mt.Wilson_4	NM58_12
1	4	3	8	3
NM58_13	NM58_35	PeruPI308523	Rivercrest_1	Rivercrest_11
4^a^	2	4	5	1
SpainW65325	SpainW65390	St.Augustine_11	St.Augustine_14	St.Augustine_3
2	4^a^	1	12	4
Starlight	TurkmenistanW62449	UzbekistanW68297		
4	8^a^	5^a^		

a Genotypes used in the no‐choice assay.

**Table A2:** List of all pairs used in the experiment

Invasive	Native
St. Augustine‐3	GIL NS 10‐6
St. Augustine‐3	Turkmenistan W62449
St. Augustine‐3	GIL NS 1‐2
St. Augustine‐3	GIL NS 4‐1
St. Augustine‐11	Hungary W65565
NM58‐12	GIL NS 10‐6
NM58‐12	CM S 12‐4
NM58‐12	Algeria W65435
NM58‐13	GIL NS 11‐4
NM58‐13	Spain W65390
NM58‐13	Turkmenistan W62449
NM58‐13	CM S 12‐4
Mt. Wilson‐4	GIL NS 10‐6
Mt. Wilson‐4	Spain W65325
Mt. Wilson‐4	Turkmenistan W62449
Mt. Wilson‐4	Hungary W65565
Mt. Wilson‐4	CM S 12‐4
Mt. Wilson‐4	GIL NS 4‐1
Mt. Wilson‐4	Algeria W65435
Mt. Wilson‐4	Malta W65375
NM58‐35	GIL NS 10‐6
NM58‐35	GIL NS 13‐6
St. Augustine‐14	GIL NS 10‐6
St. Augustine‐14	Spain W65390
St. Augustine‐14	Afghanistan PI250782
St. Augustine‐14	Greece W65319
St. Augustine‐14	Turkmenistan W62449
St. Augustine‐14	Hungary W65565
St. Augustine‐14	GIL NS 13‐6
St. Augustine‐14	CM S 12‐4
St. Augustine‐14	Algeria W65435
Starlight	Italy W65256
Starlight	Algeria W65435
Starlight	Turkmenistan W62449
Chile PI368959	GIL NS 11‐4
Chile PI368959	GIL NS 10‐6
Chile PI368959	CM S 12‐4
Brazil PI404356	GIL NS 11‐4
Brazil PI404356	GIL NS 10‐6
Brazil PI404356	Afghanistan PI250782
Brazil PI404356	Turkmenistan W62449
Australia PI197336	GIL NS 10‐6
Australia PI197336	CM NS 4‐8
Australia PI197336	Iran PI227025
Australia PI197336	Uzbekistan W68297
Australia PI197336	Greece W65319
Australia PI197336	GIL NS 1‐2
Australia PI197336	Malta W65375
Chile PI368939	Spain W65325
Rivercrest‐11	Uzbekistan W68297
Australia W65527	GIL NS 11‐4
Australia W65527	Spain W65390
Australia W65527	Uzbekistan W68297
Australia W65527	Greece W65319
Australia W65527	Turkmenistan W62449
Australia W65527	GIL NS 13‐6
Australia W65527	CM S 12‐4
Australia W65527	GIL NS 4‐1
Australia W65527	Algeria W65435
Rivercrest‐1	Afghanistan PI250782
Rivercrest‐1	Iran PI227025
Rivercrest‐1	CM S 12‐4
Rivercrest‐1	GIL NS 4‐1
Rivercrest‐1	Malta W65375
Chile PI368930	GIL NS 11‐4
Chile PI368930	Algeria W65435
Chile PI368940	Spain W65390
Chile PI368940	Iran PI227025
Chile PI368940	Uzbekistan W68297
Chile PI368940	Greece W65319
Chile PI368940	CM S 12‐4
Peru PI308523	Uzbekistan W68297
Peru PI308523	Turkmenistan W62449
Peru PI308523	Hungary W65565
Peru PI308523	Algeria W65435
Starlight	GIL NS 11‐4
Mt. Wilson‐3	Malta W65375
St. Augustine‐14	GIL NS 4‐1
St. Augustine‐14	GIL NS 1‐2
St. Augustine‐14	CMNS 5‐2
Mt. Wilson‐3	GIL NS 4‐1
Mt. Wilson‐3	CMNS 5‐2
Chile PI368930	Hungary W65565
Chile PI368940	GIL NS 10‐6
